# A co-registration investigation of inter-word spacing and parafoveal preview: Eye movements and fixation-related potentials

**DOI:** 10.1371/journal.pone.0225819

**Published:** 2019-12-18

**Authors:** Federica Degno, Otto Loberg, Chuanli Zang, Manman Zhang, Nick Donnelly, Simon P. Liversedge

**Affiliations:** 1 School of Psychology, University of Central Lancashire, Preston, United Kingdom; 2 Department of Psychology, University of Jyväskylä, Jyväskylä, Finland; 3 Academy of Psychology and Behavior, Tianjin Normal University, Tianjin, China; 4 Department of Psychology, Liverpool Hope University, Liverpool, United Kingdom; University of Valencia, SPAIN

## Abstract

Participants’ eye movements (EMs) and EEG signal were simultaneously recorded to examine foveal and parafoveal processing during sentence reading. All the words in the sentence were manipulated for inter-word spacing (intact spaces vs. spaces replaced by a random letter) and parafoveal preview (identical preview vs. random letter string preview). We observed disruption for unspaced text and invalid preview conditions in both EMs and fixation-related potentials (FRPs). Unspaced and invalid preview conditions received longer reading times than spaced and valid preview conditions. In addition, the FRP data showed that unspaced previews disrupted reading in earlier time windows of analysis, compared to string preview conditions. Moreover, the effect of parafoveal preview was greater for spaced relative to unspaced conditions, in both EMs and FRPs. These findings replicate well-established preview effects, provide novel insight into the neural correlates of reading with and without inter-word spacing and suggest that spatial selection precedes lexical processing.

## 1. Introduction

New insights on the time course of the processes underlying reading come from studies conducted with co-registration methodology. This approach involves the simultaneous recording of participants’ eye movements (EMs) and EEG signal, and provides a record of continuous brain activity over time while participants read sentences or paragraphs normally, whilst making saccadic EMs [1–9]. Compared to EM and event-related potential (ERP) techniques alone (see [1] for a discussion), co-registration allows to obtain a more fine-grain understanding of the time course of parafoveal processing, which is essential for normal reading to occur (e.g., [10–11]).

Previous research measuring readers’ eye movements has shown that completely removing spacing information or replacing it with letters, numbers, or shapes interferes with saccadic programming, produces disruption to word identification, and reduces the efficiency of parafoveal processing (e.g., [12–17]; see also [18–19]). Inter-word spaces provide visual cues of word boundaries and word length of both fixated and upcoming words (see [20–22] for reviews), and reduce lateral visual masking and visual crowding (e.g., [23–25]). Thus, when parafoveal masks do not preserve spacing information in alphabetic languages like English, reading fluency, average saccade length and skipping rate are reduced, average fixation durations, number of fixations and number of regressions increase, initial landing positions are shifted closer to the beginning of the target word (e.g., [14, 16, 24, 26]), the size of the preview effects is smaller (e.g., [27–29]), and the onset of frequency and preview effects are delayed by about 20–40 ms [16]. Sheridan and colleagues [16,29] suggested that the delayed onset for the unspaced compared to the spaced conditions might be due to less efficient parafoveal processing and slower lexical processing. If this suggestion is correct, differences between spaced and unspaced conditions should be observed both in the early and late time windows of the EEG signal, associated with parafoveal and lexical processing. To date, however, the neural correlates associated with processing of inter-word spacing information remain uninvestigated.

Likely due to the nature of the text presentation paradigms used in traditional ERP studies (e.g., rapid serial visual presentation, RSVP), the effect of inter-word spacing has never been investigated in ERP research. With respect to the FRP literature, only one co-registration experiment [9] has explored the effect of spacing, but altering the spaces between letters rather than between words. Weiss and colleagues showed voltage differences between 120–175 ms (over bilateral occipito-temporal and parietal electrode sites), 230–265 ms (over the right occipito-temporal areas of the scalp), and between 345–380 ms (over the left occipito-temporal and parietal regions) after fixation onset. The authors suggested that the effects in the time window between 120–175 ms might reflect the extraction of position-specific letter encoding and identity, and the combination of those identities into bigrams, the time window between 230–265 ms might be related to the processing of the abstract word form, and the period between 345–380 ms might be associated with higher levels of processing of the whole word. In addition, they found that an increase in foveal visual processing load (due to decreased spacing throughout the sentence) produced voltage changes between 155–220 ms after fixation onset, maximal over the occipito-temporal and parietal regions of the right hemisphere. However, as Weiss et al. pointed out, in their experiment spacing was manipulated between letters rather than between words, such that word boundaries were still well demarcated. Therefore, it remains to establish whether removing inter-word spacing produces disruption to reading with delayed onsets for even earlier aspects of reading, such as parafoveal processing.

A large body of evidence in the eye movement literature has also shown that readers extract visual and orthographic information from the parafovea (e.g., [30–33], see [20] for a review). Depending on the type of parafoveal preview, reading is more or less facilitated (see [34] for a meta-analysis). Preview benefit is calculated as the difference between processing associated with identity previews and processing associated with invalid previews. The size of the effect increases when the parafoveal mask is less ‘word-like’ (e.g., the effect is greater for an invalid parafoveal preview comprised of letters that are visually similar to those of the target word than for a preview comprised of an orthographically related word; [34]). Less word-like previews, in which visual and linguistic overlap between a parafoveal mask and a target word is minimal, produce less facilitation. Thus, it is unsurprising that when the overlap is maximal, as is the case with an identical parafoveal preview of the target word, reading times are shortest. An identity preview is estimated to speed up reading times on the target word by up to 29 ms (for first fixation duration) and 45 ms (for gaze duration) compared to previews comprised of invalid parafoveal stimuli [34]. In addition, identity previews are associated with larger saccade lengths compared to orthographically irregular or illegal previews (e.g., [35, 36]), as well as reduced numbers of fixations and regressions (e.g., [37, 38]).

A number of co-registration studies have shown that having an identical parafoveal preview of the upcoming word, compared to an invalid parafoveal preview, results in voltage differences between 140 and 300 ms after fixation onset greatest over occipito-temporal sites (known as an early *preview positivity* effect; between 140–200 ms, [39]; between 200–300 ms, [40–42]), and between 300 and 500 ms after fixation onset greatest over mid-parietal electrodes (a late *preview positivity* effect; [40–42]). In a recent study, Degno et al. [1] extended these findings in natural reading of sentences. The FRPs time-locked to the first fixation onset on the target words showed different neural correlates associated with an invalid parafoveal mask comprised of random letter strings compared to an invalid parafoveal mask comprised of strings of Xs. An X-string preview showed the early *preview positivity* effect and produced interference over a prolonged period of time (i.e., the first 500 ms after fixation onset), indicating that activation reflecting orthographic encoding and lexical identification was delayed. In contrast, a preview comprised of a random letter string led to a reduced number of voltage differences. Degno et al. argued that, although letter identities were different between the parafoveal mask and the target word, there was still feature overlap between the two stimuli (i.e., similar word shape in relation to letters with ascenders and descenders), which led to reduced disruption. In Degno et al. [1], two words in each sentence were manipulated for parafoveal preview. Thus, it remains to be established whether the neural correlates of the parafoveal preview effects that Degno et al. reported in their experiment can also be observed in different experimental paradigms, such as, for example, when multiple and consecutive words in a sentence are manipulated.

### 1.1 The present study

**Fig 1:**
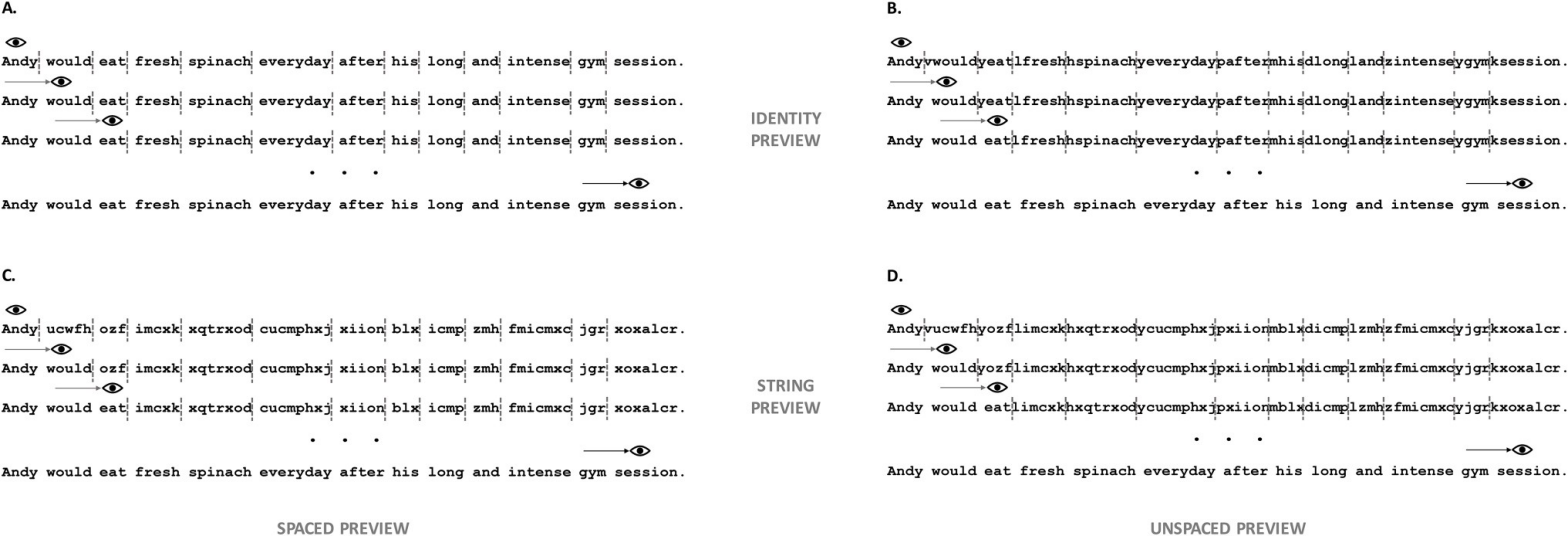
Illustration of the paradigm used. Participants read one-line sentences, and the preview of each word of the sentence was manipulated according to the boundary paradigm. Before the participants’ eyes crossed the invisible boundary, a preview was displayed in the parafovea. Panel A: the preview could be identical to the target word and with inter-word spaces intact (i.e., identity spaced preview condition). Panel B: the preview could be identical to the target word and with inter-word spaces replaced by a random letter (i.e., identity unspaced preview condition). Panel C: the preview could be comprised of a string of letters with shapes similar to the target word and with inter-word spaces intact (i.e., string spaced preview condition). Panel D: the preview could be comprised of a string of letters with shapes similar to the target word and with inter-word spaces replaced by a random letter (i.e., string unspaced preview condition).

In the present experiment we used the gaze-contingent boundary paradigm [30] to manipulate all words in the sentence for inter-word spacing (intact spaces vs. spaces replaced by a random letter) and parafoveal preview (letter string preview vs. identity preview) (see Fig 1). Participants read the single sentences for comprehension while their eye movements and EEG signal were simultaneously recorded. We aimed to investigate how the availability (unavailable vs. available) of spacing information affected processing of the parafoveal word when it was subsequently directly fixated, and to examine the neural correlates of this effect. Second, we intended to investigate how eye movements and neural correlates of parafoveal processing were influenced when incorrect (vs. correct) previews were repeatedly presented in the parafovea (i.e., successively on a word by word basis). Finally, we aimed to investigate the eye movement effects and neural correlates associated with the processing of parafoveal previews during sentence reading for spaced and unspaced text.

We decided to replace spaces with random letters to make sure that participants were not able to extract word boundary information, and thus to increase the chances of obtaining a strong effect both in the eye movement and fixation-related potential (FRP) measures (i.e., ERPs time-locked to fixation onsets). In fact, replacing inter-word spaces with numbers, shapes or with the same letter across the sentence would still produce interference due to lateral masking [23, 25], but it would be less disruptive than replacing spaces with random letters [43] as participants would very likely be able to extract word boundary information to a greater extent than in the current study.

Furthermore, we manipulated all the words in each sentence for parafoveal preview. To our knowledge, the current experiment is only the second co-registration study to investigate preview effects during normal sentence reading, and therefore, the current study may be regarded as an extension of the first. We decided to manipulate all the words in each sentence for two reasons. First, we wanted to use a paradigm that provided more data relating to foveal and parafoveal processing of a word for each experimental trial. Using the boundary paradigm for all the words in a sentence maximises the amount of data that is available per trial. Second, we wanted to demonstrate generality of effects across experimental approaches.

We expected to replicate the inter-word spacing effects in the eye movement data of the present study. Altering inter-word spaces will likely substantially influence the visual and orthographic processing of a word in the parafovea, as word length cues that may facilitate word identification will be removed, saccadic guidance will be disrupted, and processing of the parafoveal input will be less efficient. Thus, we predicted longer reading times on words that were preceded by an unspaced (in which the space was replaced by a random letter) compared with a spaced preview. With respect to the FRP results, we were less confident in our predictions, as the present experiment is the first one to investigate the neural correlates associated with the effects of inter-word spacing. However, provided that altering inter-word spacing produces disruption of visual and orthographic processing of a parafoveal word and of lexical processing of the foveal word, we anticipated increased amplitudes, as well as delayed processing associated with the more disruptive conditions. In addition, we expected to observe differences between spaced and unspaced conditions in early time windows of analysis (i.e., during those time periods associated with spillover parafoveal processing), as well as in the time windows associated with early (possibly visual and orthographic) and late (likely lexical) processing of the word in fovea.

Similarly, we expected to replicate the effect of parafoveal preview in the current experiment. We anticipated that a word preceded by a parafoveal preview comprised of a string of letters would receive longer reading times compared to a word that was preceded by an identical preview. Regarding the FRPs, we expected to replicate previous findings on the neural correlates of parafoveal preview type, that is, the early *preview positivity* effect (i.e., attenuation of the N1 amplitude for identity compared to string previews) found in experiments using flanker-word presentation, saccadic word-list reading and natural reading of sentences. With respect to the late *preview positivity* effect, it is important to note that the attenuation of negativity between 300–500 ms after fixation onset has only been observed in flanker-word presentation and saccadic word-list reading experiments. The effect has not been observed during sentence reading. For this reason, we had no strong expectation that a late preview positivity effect would occur.

Furthermore, we predicted that interactive effects could emerge both in our eye movement and FRP data, such that a more pronounced effect of parafoveal preview would likely be present in the spaced condition compared to the unspaced condition. In the unspaced condition, the letter replacing the space would produce disruption both when a word is preceded by a parafoveal preview comprised of a string of letters and when a word is preceded by an identical preview. In contrast, in the spaced condition, parafoveal processing of the preview would be disrupted only in the string preview condition.

## 2. Materials and method

### 2.1 Participants

Thirty-nine students (27 F) from the University of Southampton took part in the study. Participants’ age was between 18 and 26 years (*M* = 19.31, *SD* = 1.66). All participants were English native speakers, right handed (*M* = 84.18, *SD* = 17.30 according to the Edinburgh Handedness Inventory; [44]), with normal or corrected-to-normal vision, no reading disabilities and no history of neurological disorders. Data from one additional participant were not analysed, as their data loss was high (89 trials excluded out of 144). Participants received course credits or money for their participation.

### 2.2 Stimuli and design

We created 144 one-line sentences and asked participants to rate how plausible it was that the event in the sentence would occur. Sentences ranged in plausibility between 3.40–6.45 in a scale from 1 to 7 (1 = very implausible, 7 = very plausible; *M* = 4.88, *SD* = 0.65). All the words in each sentence were manipulated for parafoveal inter-word spacing (intact spaces vs. spaces replaced by a random letter) and parafoveal preview (preview comprised of a string of random letters vs. identical preview). However, because the fixation cross at the beginning of each trial appeared in correspondence to the first letter of the sentence, the first word never appeared as a string of letters in the parafovea. The words (that entered the analyses) ranged between 3–14 characters long (*M* = 5.67, *SD* = 1.79) and between 1.3–7.67 log Zipf frequency (*M* = 4.58, *SD* = 0.99; [45]). We used a Latin-square design so that each sentence was seen in each condition by an equal number of participants, no participant saw a different version of the same sentence twice, and each participant read an equal number of sentences in each condition. Sentences were presented according to the boundary paradigm [30]. As illustrated in Fig 1, an invisible boundary was located before the space preceding each word (or the letter filling that space in the unspaced condition) in the sentence. Before the eyes crossed the invisible boundary, a preview was displayed in the parafovea. The preview could consist of strings of letters each with a shape similar to the original letter in the word but without carrying any meaning (i.e., string condition), or it could be identical to the following fixated word (i.e., identity condition). In addition, before the eyes crossed the invisible boundary, the space between words to the right of the current fixation could be replaced by a random letter (i.e., unspaced condition), or kept intact (i.e., spaced condition). Once the participants’ eyes crossed the boundary, the preview was replaced by the correct word, and the space between words was displayed.

Originally, we also manipulated one target word in each sentence for word length (short: 3–4 characters vs. long: 6–9 characters). We selected 372 words from the English Lexicon Project [46], 186 short words (i.e., 3–4 characters long), and 186 long words (i.e., 6–9 characters long) matched for word frequency, and we paired short and long words to create the initial set of 186 sentence frames. The initial set of sentences was later reduced to match the contextual frames for predictability and plausibility between short and long target words conditions. However, due to insufficient statistical power (manipulation of only one word in the sentence for length, and eight experimental conditions), the results for this manipulation were not statistically robust and for this reason, we do not report statistical analyses associated with word length in the present paper.

### 2.3 Apparatus

The sentences were displayed on a 19-inch CRT computer screen (with a resolution of 1024 x 768 and a refresh rate of 140 Hz) and presented in black ink on a grey background, with characters in 14-point Courier New sentence case font. Sentences were displayed at a viewing distance of 70 cm, and approximately 2.19 characters subtended one degree of visual angle.

Viewing was binocular but only the movement of the right eye was recorded using a desktop EyeLink 1000 eye-tracking system, with a sampling rate of 1000 Hz. A 3-point calibration procedure was completed at the beginning of each block of sentences, and when needed during the experiment. Calibration was accepted when the average error was lower than 0.3°. Furthermore, a 1-point drift correction check was performed at the beginning of each trial.

The EEG signal was recorded from 64 scalp electrodes (Fast’n Easy Cap, Herrsching, Germany) located according to the 10–20 International system, using DC SynAmpsRT amplifiers (Compumedics Neuroscan) with a sampling rate of 1000 Hz, and it was low-pass filtered online at 100 Hz (with an attenuation of 12dB/octave). AFz was used as ground electrode, and the nose as the online reference. Additionally, four EOG channels were used to record the EEG signal associated with eye movements.

### 2.4 Procedure

The present study was approved by the Ethics Committee of the University of Southampton (ERGO number: 25066) and all participants gave written informed consent before taking part in the experiment. At the beginning of the experimental session, three tests were conducted to make sure that our participants met the inclusion criteria of the study. First, they were asked to fill the Edinburgh handedness inventory [44], to confirm that participants were right handed. Second, they were tested for visual acuity with the Landolt “C” eye chart (Precision Vision, La Salle, United States), to ensure that participants met 20/20 vision at 4 m viewing distance. Third, a calibration procedure was performed, to check that participants’ eyes could be accurately tracked. All participants who successfully passed the three tests, took part in the experiment.

The experimental session involved a first block of 10 practice sentences, and four blocks comprised of 36 experimental sentences, which were presented in a random order for each subject. Participants were asked to silently read each sentence and to answer comprehension questions for 25% of the trials, while their eye movements and EEG signal were simultaneously recorded.

Each trial began with a cross on the left side of the screen. Participants were required to fixate the cross for 500 ms, after which the fixation cross was replaced by the first letter of the sentence. Participants were instructed to silently read the sentence and to fixate on a cross on the right side of the screen to terminate the current trial and initiate the following one.

The experimental session lasted about one hour, and participants were given the opportunity to take breaks at the end of each block of sentences or at any point if needed.

### 2.5 Co-registration of eye movements and EEG signal

A marker was sent at the onset and offset of each trial from the display computer (running SR Research Experiment Builder) to the computers recording the EEG signal and eye movements. An offline synchronization of eye movements and EEG signal was then performed via the EYE-EEG extension of EEGLAB toolbox [2]. Deviations between the markers of both recordings were equal or shorter than 2 ms in absolute value (*M* = 0.35, *SD* = 0.48), confirming the good quality of the synchronisation.

### 2.6 Pre-processing of eye movement and FRP data

We analysed eye movement and FRP measures associated with each word of the sentence to provide details of the on-line time course of processing during first-pass reading. We used SR Data Viewer to select only fixations longer than 50 ms and shorter than 800 ms to be entered in the analyses, as well as those fixations where there was no early or late display change (i.e., a display change during a fixation on the pre-boundary word, or when the display took more than 10 ms after the fixation onset on the post-boundary word to change), hooks, blinks and/or skips. Moreover, only consecutive fixations, which landed first on word N-1 and then proceeded onto word N, and fixations that occurred during first-pass reading were analysed. In addition, only words equal or longer than three characters, and only those words not in the first or last position within the sentence were entered into the analyses.

We used the EEGLAB 14_1_1b [47] toolbox for Matlab (version R2015a) to band-pass filter the EEG data offline, with a high-pass band edge frequency of 0.1 Hz and a low-pass band edge frequency of 30 Hz. The extended Infomax Independent Component Analysis (ICA) algorithm [48] was performed to identify the ocular artefacts first on trained segments band-pass filtered between 1–30 Hz, and then on epochs of 900 ms (-100 ms to +800 ms from fixation onset). The independent components associated with ocular artefacts were identified according to the EYE-EEG extension [2], and pruned from the data as oculomotor artefacts (*M* = 2.62, *SD* = 0.94) if they shared temporal covariance higher than 1.1 with eye movements [49]. In addition, if the difference between minimum and maximum voltage at each scalp electrode was greater than 150 μV (in absolute value) for more than 5% of all the EEG segments, then spherical interpolation of that channel was performed, which occurred for eight of our participants. Thereafter, the EEG signal was re-referenced against the average of all scalp electrodes and baseline-corrected by subtracting the 100 ms preceding each fixation onset. Furthermore, epochs with extreme values (i.e., greater than 120 μV in absolute value) in any scalp channel were excluded from the analyses. Lastly, FRPs were averaged within and then across participants for analyses.

The final dataset included 26,428 observations in total, with an average of 169.41 observations per participant per condition (*SD* = 47.24, range = 29–273).

### 2.7 Eye movement and FRP statistical analyses

We used the “lmer” function from the lme4 package [50] within the R framework for statistical computing [51] to run linear mixed effects models on log transformed first fixation duration (the duration of the first fixation on a word during first-pass reading), single fixation duration (the duration of the only fixation made on a word during first-pass reading) and gaze duration (the sum of all fixations on a word during first-pass reading, before readers fixate another region). All findings reported here are from models including a full random structure for both subjects and items (with both random intercepts and slopes), as per Barr, Levy, Scheepers and Tily [52]. To set up the levels of each fixed factor (i.e., inter-word spacing and parafoveal preview), we used the function “contr.sdif” from the MASS package (version 7.3–45; [53]). The p-values were estimated using the “lmerTest” package (version 2.0–32; [54]).

The analyses of the FRPs were conducted over four time windows: between 0–70 ms, 70–120 ms, 120–300 ms and 300–500 ms. The earliest time-window was chosen to investigate the very early effects spilling over from the parafovea onto the currently fixated word. The following two time-windows were chosen to investigate early components that are known to show effects of visual and orthographic manipulations, namely, P1 and N1 components, and a later component that typically shows effects associated to lexical (and post-lexical) access, that is, the N400 component. To examine the neural correlates of the inter-word spacing and parafoveal preview effects, we analysed the FRP epochs time-locked to fixation onset for each word in the sentence.

FRPs were analysed with a two-tailed non-parametric cluster-based permutation test [55] using the Matlab toolbox *FieldTrip* [56]. By performing this type of analysis we were able to examine the electrical activity at each of the multiple electrode sites and time points of our time windows of analysis, but controlling for the multiple comparisons problem [55, 57, 58]. Hence, we computed 10,000 iterations to generate the permuted data and we included in the test each scalp channel and each time point within each of the time windows considered. For a selected sample (i.e., channel-time pair) to be included in the clustering algorithm, at least two neighborhood channels were required to be significant. The observed two-tailed cluster-level t-statistic was considered significant when the *p* value was less than 0.025 in each tail.

In order to conduct cluster-based permutation analyses on the interaction between inter-word spacing and parafoveal preview, we calculated voltage differences between string and identity previews in both the unspaced and spaced conditions. For each of the time windows of analysis, if the interaction term reached significance, we carried out the pairwise comparisons.

## 3. Results

### 3.1 Accuracy

The average accuracy to comprehension questions was 96% (*SD* = 4.11), showing that participants read and understood the sentences.

### 3.2 Display change awareness

Out of the thirty-nine participants, all participants reported having noticed that something unusual occurred on the display (thirty-five participants reported it spontaneously, and four participants after being informed of the changes). Participants estimated on average that 47.54 display changes (*SD* = 37.85) occurred (in fact display changes actually occurred in the experiment for each word in 108 sentences). Twenty-seven participants perceived that words in the sentence were replaced by “jumbled” words, whilst eighteen participants noticed that spaces between words were removed. This finding is consistent with a number of studies in the literature [59, 60] reporting that participants are able to detect changes when presented with visually unusual previews.

### 3.3 Eye movements

We observed a significant effect of inter-word spacing (see Table 1), such that reading times were significantly shorter when the parafoveal preview preserved the spaces between words compared to when the spaces were replaced by letters (difference of 30 ms in first fixation duration, 35 ms in single fixation duration, 98 ms in gaze duration). These findings confirm previous results reported in the literature (e.g., [14, 24]), and suggest that replacing spaces with letters produces early disruption to eye guidance and word identification, and reduces the efficiency of parafoveal and foveal processing.

**Table 1:** Model parameters, observed means and standard deviations for eye movement data analyses.

		Model				Condition			
		*b*	SE	*t*	Sig.	Identity Spaced	String Spaced	Identity Unspaced	String Unspaced
		Local Analysis							
FFD	Intercept	5.505	0.015	370.460	*	221 (69)	267 (79)	268 (85)	280 (87)
	Space	-0.122	0.011	-11.140	*				
	Viewing	0.120	0.008	15.210	*				
	Space*Viewing	0.150	0.013	11.800	*				
SFD	Intercept	5.522	0.016	340.710	*	220 (68)	274 (76)	274 (84)	289 (91)
	Space	-0.136	0.012	-11.300	*				
	Viewing	0.141	0.010	14.500	*				
	Space*Viewing	0.174	0.015	11.770	*				
GD	Intercept	5.669	0.018	312.130	*	244 (97)	300 (104)	361 (181)	378 (184)
	Space	-0.274	0.014	-20.020	*				
	Viewing	0.14	0.011	13.280	*				
	Space*Viewing	0.171	0.017	10.330	*				

Means and standard deviations have been calculated by subjects. FFD = first fixation duration, SFD = single fixation duration, GD = gaze duration.

* *p* < .05.

A significant effect of parafoveal preview was also observed on all the analysed measures. First pass reading times were significantly shorter for those words preceded by an identical preview relative to those words that were preceded by a preview comprised of a string of random letters (difference of 29 ms for first fixation duration, 35 ms for single fixation duration, 37 ms for gaze duration). These results confirm previous findings in the literature and provide further evidence that having a valid parafoveal preview facilitates word identification ([22]), and that facilitation is evident already from first pass reading.

Finally, we observed significant interactive effects on first fixation duration (46 ms vs. 12 ms), single fixation duration (54 ms vs. 15 ms), and gaze duration (56 ms vs. 17 ms). These findings support previous results (e.g., [27, 28]) indicating that the size of the preview effect is larger for spaced relative to unspaced conditions.

### 3.4 Fixation-related potentials

The results from the cluster-based permutation analyses (see Table 2 for a summary) are consistent with the EM data.

**Table 2:** Summary of the statistical differences between conditions observed in the frp data with cluster-based permutation tests.

Comparison	Time Window							
	0–70 ms		70–120 ms		120–300 ms		300–500 ms	
	Cluster	p-value	Cluster	p-value	Cluster	p-value	Cluster	p-value
String—Identity	positive	ns	positive	<.002	2 positive	<.001	positive	<.001
						<.003		
	negative	ns	negative	<.02	2 negative	<.001	negative	<.001
						<.002		
No Space- Space	positive	ns	positive	<.003	2 positive	<.001	positive	ns
						<.001		
	negative	<.005	negative	<.001	negative	<.001	negative	ns
Viewing*Spacing	positive	ns	positive	ns	2 positive	<.001	positive	<.02
						<.01		
	negative	ns	negative	<.003	2 negative	<.001	negative	<.001
						<.01		
StNS-IdNS	NA	NA	NA	NA	positive	<.001	positive	ns
	NA	NA	negative	ns	2 negative	<.002	negative	ns
						<.02		
StS-IdS	NA	NA	NA	NA	2 positive	<.001	positive	<.001
						<.001		
	NA	NA	negative	<.01	2 negative	<.001	negative	<.001
						<.001		

Viewing*Spacing was calculated as the difference between (StNS—IdNS) and (StS—IdS). StNS: String No Space, IdNS: Identity No Space, StS: String Space, IdNS: Identity No Space. NA: no cluster–based permutation test was conducted.

**Fig 2:**
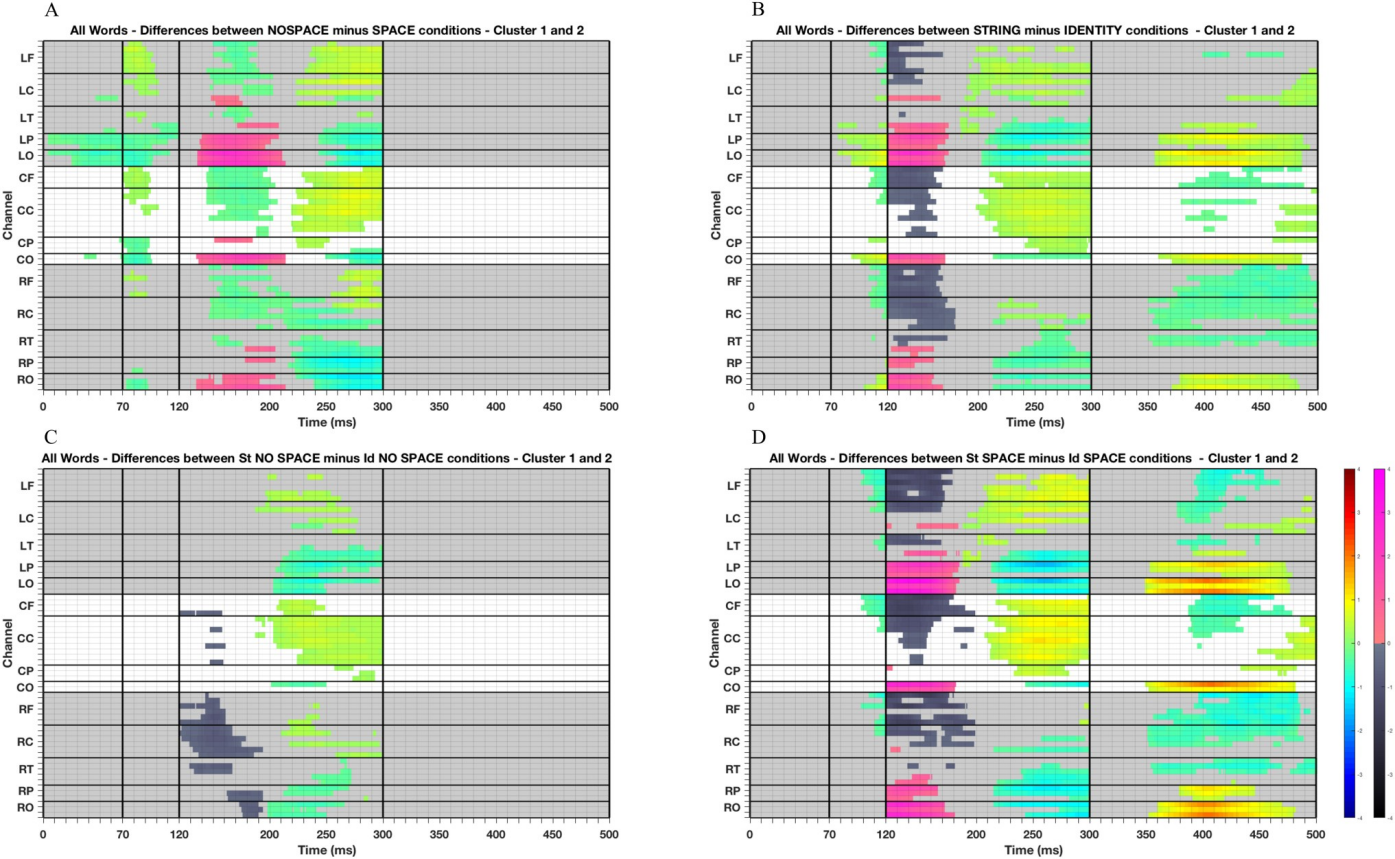
Raster diagrams illustrating significant FRP differences obtained with cluster-based permutation tests. Red and blue rectangles indicate the channel/time point in which the first condition was significantly more positive or negative than the second condition, respectively, in cluster 1. Pink and black rectangles indicate the channel/time point in which the first condition was significantly more positive or negative than the second condition, respectively, in cluster 2. Channels are displayed on the y-axis and organised somewhat topographically. Channels on the left hemisphere (L) of the scalp are shown in the figure’s top grey rectangle and demarcated with horizontal lines based on their location in the frontal (LF: FP1, AF7, AF3, F7, F5, F3), central (LC: FC5, FC3, C5, C3, CP5, CP3), temporal (LT: FT9, FT7, T7, TP9, TP7), parietal (LP: P7, P5, P3) and occipital (LO: PO7, PO3, O1) regions of the scalp. Midline electrodes (C) (i.e., CF: FPz, F1, Fz, F2; CC: FC1, FCz, FC2, C1, Cz, C2, CP1, CPz, CP2; CP: P1, Pz, P2; CO: Oz, POz) are displayed in the middle. Right channels (R) are shown on the figure’s bottom grey rectangle (i.e., RF: FP2, AF8, AF4, F8, F6, F4; RC: FC6, FC4, C6, C4, CP6, CP4; RT: FT10, FT8, T8, TP10, TP8; RP: P8, P6, P4; RO: PO8, PO4, O2). The time from the onset of a fixation on the word is displayed on the x-axis. The vertical black lines indicate the time windows considered for the cluster-based permutation tests: between between 0–70 ms, 70–120 ms, 120–300 ms, 300–500 ms. (A) FRP differences between previews in which the spaces were replaced by a random letter and previews in which spaces were kept intact, (B) between pretarget words with previews made of a string of letters and identity previews, (C) between previews made of a string of letters and identity previews both in which the spaces were replaced by a random letter, (D) between previews made of a string of letters and identity previews both in which the spaces were kept intact.

**Fig 3:**
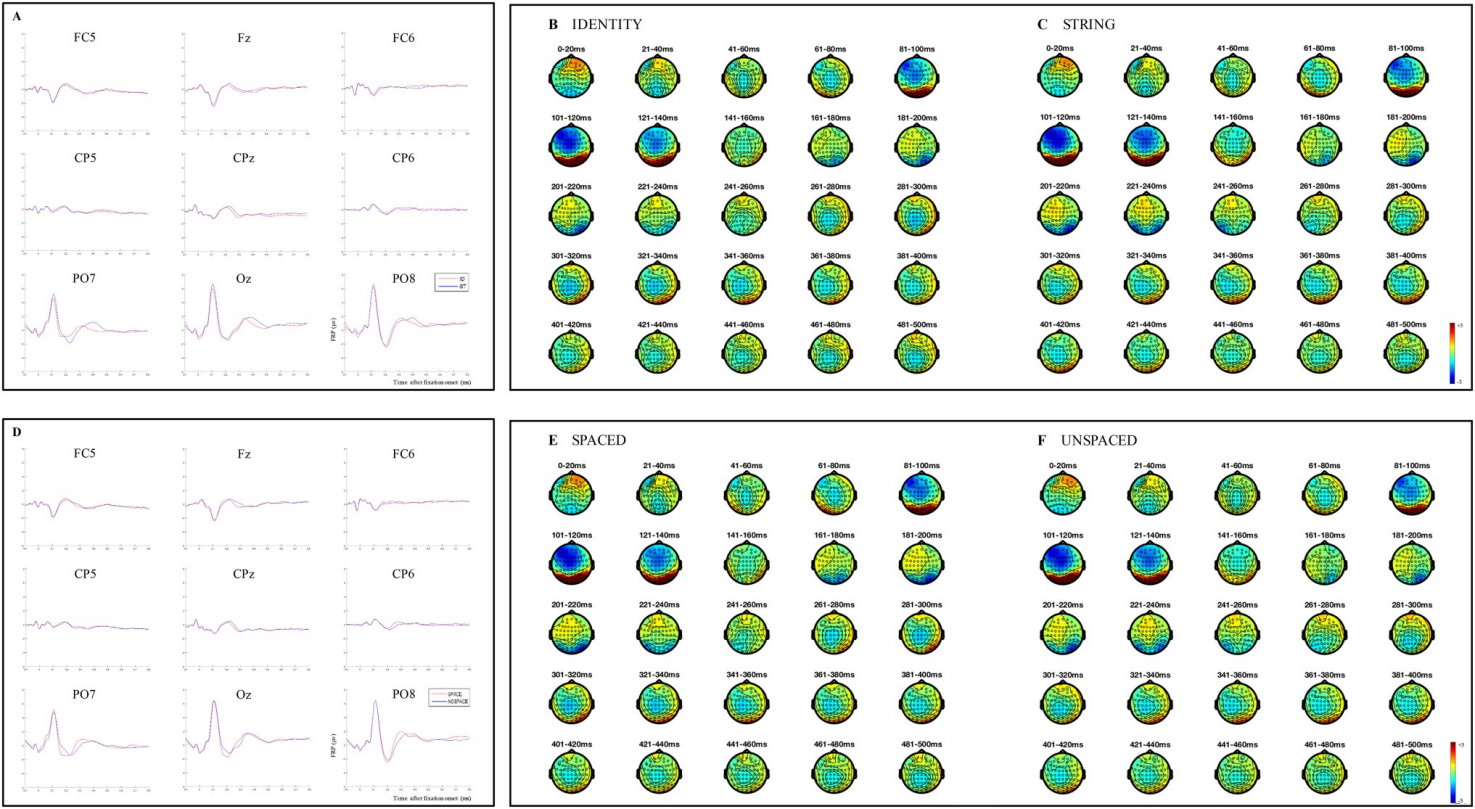
Results from FRPs time-locked to the fixation onset on each word. (A) Grand average FRPs in response to the ID (i.e., identical preview) and ST (i.e., random letter string preview) conditions displayed on the same nine channel locations as in Panel A. (B) Topographies showing the average brain activity associated with the ID condition for 20-ms time windows from 0 to 500 ms after fixation onset. (C) Topographies showing the average brain activity associated with the ST condition for 20-ms time windows from 0 to 500 ms after fixation onset. (D) Grand average FRPs in response to the SPACED (i.e., conditions in which the spaces displayed in the parafovea were kept intact) and UNSPACED (i.e., conditions in which the spaces displayed in the parafovea were replaced with a random letters) conditions displayed on nine channel locations, left frontal (FC5), midline frontal (Fz), right frontal (FC6), left centro-parietal (CP5), midline centro-parietal (CPz), right centro-parietal (CP6), left parieto-occipital (PO7), midline occipital (Oz), right parieto-occipital (PO8) electrodes. (E) Topographies showing the average brain activity associated with the SPACED condition for 20-ms time windows from 0 to 500 ms after fixation onset. (F) Topographies showing the average brain activity associated with the UNSPACED condition for 20-ms time windows from 0 to 500 ms after fixation onset.

Between 0–70 ms, we observed negative differences between unspaced and spaced preview conditions over the left occipital and parietal areas of the scalp, as well as differences at single neighbour electrodes over the left central, left temporal, central occipital and parietal sites of the scalp (see Fig 2A). These differences sustained into the following time window, between 70–120 ms, extending to central parietal and right occipital and parietal electrodes. In this window of analysis, positive differences between unspaced and spaced preview conditions were also observed, over frontal regions, which might reflect opposite electrical field potential (i.e., more negative activation at the posterior regions of the scalp and more positive at the front for the unspaced condition), and over left central and temporal, and mid-central electrodes. In Fig 3D–3F, the waveforms and topographies corresponding to the time windows 0–70 ms and 70–120 ms indicate that the positive activation over posterior regions of the scalp (i.e., the P1 component) occurred earlier for the spaced compared to the unspaced condition. Differences in these time windows and over posterior areas of the scalp, are typically associated with spillover parafoveal processing, and visual processing of a word (e.g., [61]). Hence, the statistically significant differences together with the FRP topographies suggest that in the spaced conditions readers were able to process word boundaries, and therefore identify which letters comprised the following word to be processed. This more efficient parafoveal pre-processing for the spaced condition allowed visual encoding of the fixated word to proceed normally for the spaced but not for the unspaced conditions.

Between 120–300 ms, we observed two separate significant clusters of positive differences and one significant cluster of negative differences between unspaced and spaced preview conditions (see Fig 2A). Here, as well as later in the manuscript, we will describe the significant clusters in the temporal order in which they appear. However, note that the temporal order does not reflect the statistical weight of significance. The first, in terms of temporal order, cluster of significant differences was always statistically significant, but less robust, than the second cluster. The first cluster of positive differences is observed between about 130–200 ms, greatest over occipital and left parietal regions of the scalp, but showing some differences also over temporal, left central, right and mid-parietal scalp electrodes. The second cluster of positive differences is seen between about 220–300 ms mainly over frontal and central areas of the scalp, as well as over mid-parietal electrodes. The negative differences are observed first over frontal, central and temporal electrodes, and subsequently over occipital, and parietal regions. Fig 3D–3F show that the N1 component is delayed for the unspaced compared to the spaced conditions. Differences between 120–300 ms and over occipital, parietal and temporal electrodes are considered to reflect orthographic (and possibly phonological) activation of a word (e.g., [40]). Thus, these findings, taken together with the FRP topographies, suggest that in the unspaced condition orthographic processing cannot proceed at a normal pace and as effectively as in the spaced condition. This causes both a delay in the start of the N1 component and a protraction of the orthographic processing associated with the unspaced condition. In contrast, in the spaced condition, orthographic processing can proceed normally and trigger higher levels of processing (as possibly shown by the start of the N400 component from about 261–280 ms onwards). In fact, in the spaced condition, it appears that the N1 component is strongest in the initial period of this time window, over parietal and occipital regions of the scalp, and this activation then dissipates forming a more central negativity in the later part of this time window.

With respect to the presence of two clusters within the same time window of analysis and with the same direction of the effect (i.e., two positive and/or two negative clusters) rather than a single cluster, this result was unexpected on the basis of the existing FRP literature (e.g., [9]), in which only single significant clusters have been reported. It is possible that the presence of two clusters here might be due to the nature of the identification procedure we (and others) adopted in the cluster-based permutation analyses. Spatio-temporal clusters are identified when similar effects occur over at least two adjacent time-electrode samples. Therefore, under specific circumstances, when even very small non-significant gaps (in terms of time and/or electrodes) occur, separate and independent significant clusters might be identified, rather than a single unified cluster. Our FRP data show a very limited gap, approximately between 215–220 ms, during which no significant difference is found. Such effects may reflect two possible situations. One possibility is that the two clusters might reflect two different stages (or aspects) of processing, likely to be orthographic processing for the first cluster, and phonological processing or lexical access for the second cluster. This interpretation might fit well with the results observed in foveal masked priming experiments, where an N/P150 component, assumed to reflect letter-to-word form processing, was detected between 90–200 ms, and an N250 component, considered to reflect letter-to-whole-word form processing, was detected with a peak around 250 ms [62]). If the two clusters reflect different stages of processing, it will be critical for future research to establish the precise experimental conditions that lead to one versus two significant clusters. A second, arguably, less theoretically interesting hypothesis is that the unexpected presence of the two clusters might reflect the same underlying stage of processing, but the differences failed to maintain significance during the entire period of analysis, albeit for a very limited temporal window of approximately 5 ms.

Regarding the effect of parafoveal preview, the earliest statistical differences occurred in the time window between 70–120 ms. We observed a positive cluster between string and identity preview conditions at all electrodes of occipital and left parietal areas of the scalp, as well as at single electrodes over left temporal and central electrodes, and centro-parietal sites. A negative cluster was also observed, over left frontal, left central, midline frontal and central, right frontal and central (and right temporal for a few milliseconds) areas of the scalp. The waveforms and topographies provided in Fig 3A–3C indicate that the P1 component was stronger in this time latency for the string compared to the identity preview conditions, suggesting that visual processing was still being carried out for the invalid, but less so for the valid preview condition.

As for the results of inter-word spacing, between 120–300 ms we observed multiple significant clusters, two for positive and two for negative differences between string and identity preview conditions. The earliest significant cluster of positive differences appears maximal over occipital, and both right and left temporal and parietal regions, while a significant cluster of negative differences is observed greatest over central, and frontal sites. The subsequent cluster of positive differences is seen maximally over central, left and midline frontal and mid-parietal regions, and the second cluster of negative differences over occipital, and both right and left occipital, temporal and parietal areas. As explained for the previous findings, the presence of multiple clusters might reflect different cognitive processing (e.g., orthographic and phonological processing or lexical access), or be due to gaps of time during which none of the differences reaches significance at any of the electrodes (here from about 175–185 ms for the positive differences, and between 180–200 ms for the negative differences). When considering the FRP waveforms and topographies (see Fig 3A–3C), it appears that significant differences between 120–300 ms can be explained by the time course of the N1 component, which was stronger and more extended for the string compared to the identity preview conditions.

Finally, between 300–500 ms, a positive cluster was observed to be maximal over occipital and left parietal and central areas of the scalp, while negative differences were observed greatest over right temporal, central and frontal regions. Likely due to the more prolonged N1 component in the string preview condition, the N400 component started later in the string compared to the identity preview condition between 300–500 ms. Thus, the present FRP data associated with the N1 and N400 components show both the early [1, 39–42] and, to some extent, the late *preview positivity* effects [40–42], and suggest an earlier start for the orthographic and lexical processing associated with the identity preview conditions relative to the string preview conditions. We note however, that we do not observe large and widespread centro-parietal differences typically associated with the late *preview positivity* effect on the N400. Instead, in this time window, we observe larger voltage differences over the occipital channels (differences that become even larger when string and identity preview spaced conditions are compared, see Fig 2B and 2D). Nevertheless, the occipital pattern might still be part of the late N400 *preview positivity* effect and reflect opposite electrical field polarity to that observed over the more classic N400 scalp distribution.

**Fig 4:**
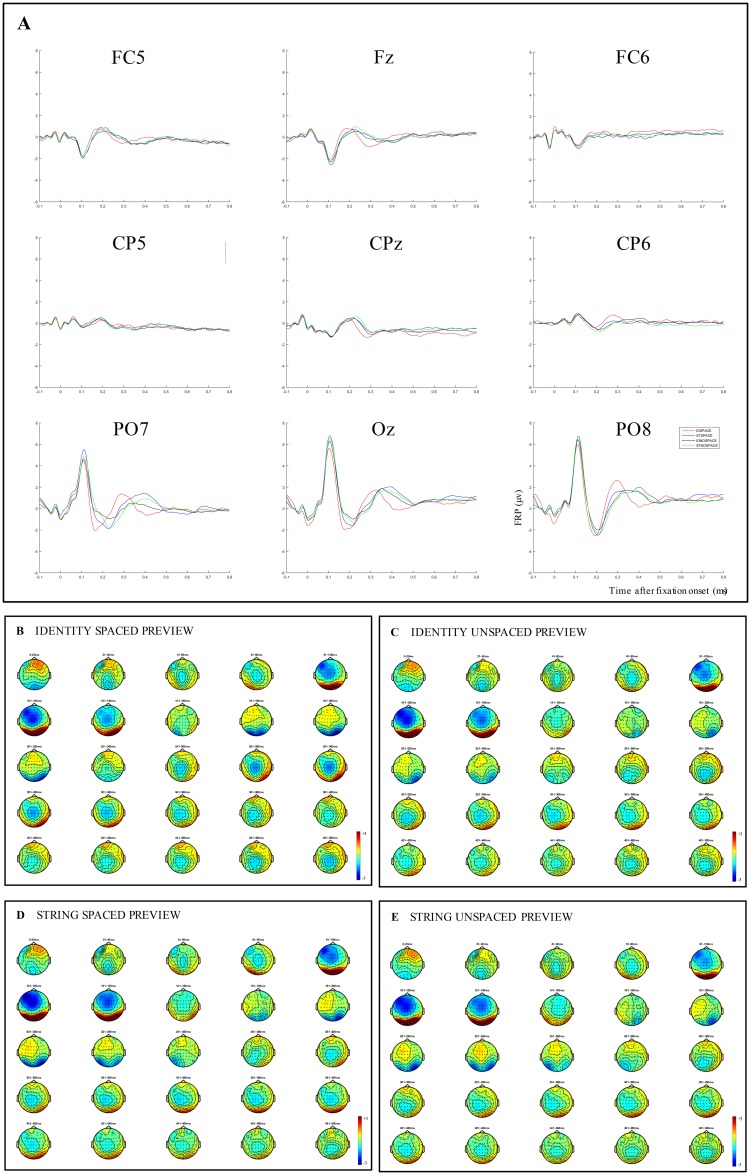
Results from FRPs time-locked to the fixation onset on each word. (A) Grand average FRPs in response to the ID SPACED (i.e., identical and spaced previews), the ST SPACED (i.e., random letter string and spaced preview), the ID UNSPACED (i.e., identical previews with the spaces, as displayed in parafovea, replaced with a random letter), the ST UNSPACED (i.e., random letter string preview with the spaces, as displayed in parafovea, replaced with a random letter) conditions displayed on nine channel locations, left frontal (FC5), midline frontal (Fz), right frontal (FC6), left centro-parietal (CP5), midline centro-parietal (CPz), right centro-parietal (CP6), left parieto-occipital (PO7), midline occipital (Oz), right parieto-occipital (PO8) electrodes. (B)-(E): Topographies showing the average brain activity associated with each condition for 20-ms time windows from 0 to 500 ms after fixation onset.

Consistent with our predictions, we also observed significant interactive effects. The FRP data confirmed that there was a smaller preview benefit in the unspaced conditions relative to the spaced conditions. The only significant differences between string and identity in the unspaced conditions were observed in the time window between 120–300 ms after fixation onset (see Fig 2C). Waveforms and topographies displayed in Fig 4A, 4C and 4E show that the differences observed in this time window of analysis can be attributed to differences in the N1 component, which appears to be stronger and to extend for about 20 ms longer in the string unspaced condition, compared to the identity unspaced condition. From the cluster-based permutation tests, we can see that two clusters of negative differences and one cluster of positive differences reached significance. The first cluster of negative differences was observed mainly over right frontal, central, temporal, parietal and occipital areas of the scalp, and over single electrodes of midline-central and frontal areas.

There are some similarities between this cluster and the effects observed for the main effect of parafoveal preview, and for the parafoveal preview effect under the spaced conditions, in terms of areas and time where the differences reached significance. However, there are also some differences. Here, no positive differences were observed between approximately 120–200 ms, no negative difference reached significance over the left hemisphere, and only very limited electrodes of the central areas of the scalp showed a negative difference. Given that this is the first study to investigate neural correlates of parafoveal processing with unspaced text, it is not entirely clear what processing this first cluster might represent. However, as we stated earlier, the presence of two clusters might simply be due to a small number of time points in which no significant effect was found over adjacent electrodes. Thus, the two clusters might represent the same underlying processing (likely orthographic processing), or they might represent different levels of processing (e.g., orthographic and phonological, or lexical, processing). Clearly, further work is required to adjudicate between the possibilities.

The second cluster of negative differences was observed mainly over occipital, parietal and temporal areas of both left and right hemispheres. Nearly aligned to this cluster, significant positive differences were observed over frontal and central, as well as centro-parietal electrodes. We note that the second cluster of negative and the cluster of positive differences appear very similar to the results obtained for the main effect of parafoveal preview, and for the parafoveal preview effect under the spaced conditions. These two clusters resemble the early *preview positivity* effect, and suggest that facilitation occurs when readers successfully pre-processed the correct orthographic word form in the parafovea.

With respect to the spaced conditions, the cluster-based permutation analyses reveal very comparable, but larger, differences from the main effect of parafoveal preview (see Fig 2D). Similar to the main effect of parafoveal preview, negative differences between string and identity preview spaced conditions were observed in the time window between 70–120 ms, over frontal and central areas of the scalp, as well as over left temporal electrodes. However, different relative to the main effect of parafoveal preview, no significant positive differences reached significance here in this time window of analysis.

Between 120–300 ms we found two clusters of positive and two clusters of negative results. The earliest cluster of negative differences between string and identity preview spaced conditions is seen maximally over frontal, central and temporal regions, and the earliest cluster of positive differences over left, right and midline occipital areas, over left and right parietal sites, and over single electrodes in the left central and left temporal, central-parietal, right central and temporal regions. Similarly, the second significant cluster of negative differences in this time window appears maximal over occipital, temporal, and right and left parietal regions, while a significant cluster of positive differences is observed mainly over left frontal, central, temporal and parietal areas, as well as over midline frontal, central and parietal sites. Again, these differences resemble very closely the differences found in relation to the main effect of parafoveal preview, but the size of the difference is larger here. When considering the FRP waveforms and topographies (see Fig 4A, 4B and 4D), it appears that significant differences in this time window can be explained by the time course of the N1 component, which started and dissipated earlier for the identity compared to the string preview conditions.

In the following time-window, between 300 and 500 ms after fixation onset, a positive cluster was observed to be maximal over occipital, as well as left and right parietal areas of the scalp, while negative differences between string and identity preview spaced conditions were observed over right and left temporal, as well as over central and frontal regions. These differences might be explained by the time course of the N400 component, which started about 40 ms later in the string compared to the identity spaced preview conditions. Again, here we observe both the early [1,39–42] and, to some extent, the late [40–42] *preview positivity* effects. Our data seem to confirm that, in the spaced conditions, a valid preview leads to more efficient processing, both with respect to orthographic and lexical stages of processing.

## 4. Discussion

### Inter-word spacing

One of the aims of our experiment was to investigate for the first time the neural correlates associated with processing of inter-word spacing. We hypothesised that removing spaces between words could disrupt pre-processing of the word in parafovea at a visual and orthographic level, and processing of the word in fovea at the lexical level. Hence, we anticipated to find eye movement results similar to the ones reported in the existing literature, such that reading times are shorter for spaced than unspaced conditions (e.g., [24]). In addition, we anticipated to find a difference in the FRP data between spaced and unspaced conditions in early time windows (possibly between 0–300 ms), associated with spillover effects from the parafovea, as well as with visual and orthographic processing, and in a later time window (between 300–500 ms) associated with effects of lexical manipulations.

Our EM data confirm previous findings indicating that filling spaces between words with random letters disrupts eye guidance of the next eye movement as well as word identification of the fixated word. In the unspaced condition, readers need to fixate longer in order to verify their understanding of the text and to resolve difficulty associated with reading of unspaced text. These changes in eye movement behaviour serve to allow readers to form a full and comprehensive understanding of the sentence meaning. The FRP data extended these results, showing that altering inter-word spaces affects very early stages of processing. Based on our FRP findings, we argue that replacing spaces with random letters disrupts and delays parafoveal processing of word N+1, as well as (at least) visual encoding and activation of the orthographic (and likely phonological) representation of the fixated word. Interestingly, we observe an attenuation of the N1 component for the spaced compared to the unspaced condition in the time window between 120–300 ms after fixation onset. This result seems to resemble the early *preview effect* previously found in ERP/FRP experiments investigating different types of invalid parafoveal preview, such as other semantically related or unrelated words, as well as strings of Xs [1,39–42]. To find a similar effect associated with inter-word spaces might suggest that the *preview positivity* effect could reflect not only activation of the orthographic representation of a word, but it could also be an index of delayed or disrupted stages of orthographic processing (i.e., when a parafoveal preview is manipulated in such a way that orthographic processing cannot proceed at a normal pace or as effectively).

In the latest time window of analysis, between 300–500 ms (i.e., the N400 component latency range), none of the differences between unspaced and spaced preview conditions as observed in the FRP topographies reached significance in the permutation analysis. Assuming that between 300–500 ms effects associated with lexical processing start to emerge, and that the inter-word spacing manipulation affects both eye guidance and word identification, then we might have anticipated significant differences here (e.g., [12, 29]). A possible explanation for lack of effects may be that the string spaced preview conditions showed an activation very similar to both the string and identity unspaced conditions (see Fig 4), and this might have cancelled out the effect on this time window. To be clear, the experimental manipulations were such that effective processing of words in the parafovea was not possible in three (i.e., identity unspaced, string unspaced, string spaced previews) out of four of the experimental conditions. Replacing spaces with random letters meant that identifying the letters comprising the next word was almost impossible. Furthermore, effective processing of an upcoming word was prevented with a random letter string preview. Under these circumstances, and as we observed, one might anticipate that effects would be comparable for these three conditions relative to the identity preview condition.

### Parafoveal preview

Another objective of the current study was to examine neural correlates of parafoveal preview in natural reading. Only one other experiment has investigated this effect during reading of sentences [1]. In that experiment, two words in each sentence were manipulated for parafoveal preview, which could be comprised of a string of Xs, or a string of random letters, or which could be identical to the target word. Because both experiments used parafoveal previews comprised of a string of random letters and previews identical to the target word, one might expect the current study to produce results for these conditions that are comparable to those obtained in Degno et al. [1]. However, as discussed in the Introduction of this paper, differences in experimental design (i.e., two single manipulated words within a sentence versus all of the words in the sentence being manipulated) are crucial when considering the results of the two studies in relation to each other. First, the behavioural data from both experiments show that the previews comprised of a string of letters appeared quite natural in the parafovea in Degno et al. [1], as only few participants detected such previews, whilst previews appeared quite unusual in the current experiment, as almost all participants detected this type of preview manipulation. In this sense then, the string of random letters in the current experiment can be considered more similar to the preview condition with Xs used by Degno et al., which was detected by all participants. Second, due to the nature of the experimental manipulation used in the present study, a different baseline had to be used here compared to Degno et al. In the current experiment, we used as baseline period the 100 ms preceding the fixation onset of each word that entered the analyses (same baseline as, for example, in [39, 41]). In contrast, in Degno et al. the baseline was the 100 ms preceding the fixation onset of the pre-target word (for FRPs time-locked to both pre-target and target fixation onsets). Certainly, when all the words in a sentence are manipulated, it is more difficult to find a baseline which does not contain pre-existing differences at the event of interest (i.e., the fixation onset), compared to when only a few words are manipulated and the words preceding and following each target word are identical across conditions. In this respect, the present experiment is much more similar to previous experiments that have used saccadic word-list reading (e.g., [39–41]).

Our EM data provided evidence that a parafoveal preview facilitates processing upon fixation of that word. Interestingly, the effect size of inter-word spacing was larger compared to the size of the parafoveal preview effect. This may indicate that when it is not possible to extract word boundaries from the parafovea, as is the case in the unspaced condition, it is more difficult to recover from disruption. Whereas when extraction of letter identities from the parafovea is incorrect, as is the case in the string preview conditions, some letter features can still be activated (e.g., shape of the word), and these features might disrupt reading to a lesser degree and recover can occur more rapidly, once the word is fixated.

The FRP data extended further the EM results. They showed that in our experiment, where all the words in the sentence were manipulated, the effect of parafoveal preview elicited differences that started slightly later than the effect of inter-word spacing (significant differences of parafoveal preview conditions between 70–500 ms, significant differences of inter-word spacing conditions between 0–300 ms). These results fit well with the suggestion that spaces and letters are simultaneously attended to and processed in the parafovea, but spaces are processed faster than letters, because they are initially processed more automatically at a basic visual level. Such processing occurs with a more immediate time course than orthographic processing of letter form and identity [63].

Furthermore, as mentioned earlier, we note that when comparing these results with the existing literature, the current findings appear more similar to the findings observed in saccadic word-list reading and experiments with flanker-word presentation, rather than sentence reading. Indeed, differences between 0–70 ms were observed during reading of sentences [1], both between X-string and identity previews, and strings of letters and identity previews, but such differences were not investigated in previous saccadic word-list reading experiments [39–42], and are not found in the current experiment. In contrast, an attenuation of the N400 negativity was observed over centro-parietal sites in saccadic word-list reading [40–42] and, to some extent, in the current study, but not during sentence reading [1]. One could speculate that such differences might be due to the naturalness of the task in which the participant is engaged. However, it remains for future research to establish whether this is the case, and beyond this, the role that different baselines play in modulating neural correlates of parafoveal preview type (see also [64]).

### Interactive effects

We also aimed to investigate the neural correlates associated with the processing of parafoveal previews during sentence reading under conditions of spaced and unspaced reading. We expected the effect of parafoveal preview type to be larger in the spaced compared to the unspaced conditions. The rationale here was that when the spaces in the parafovea are filled with random letters, word boundaries cannot be extracted, and both eye guidance and word identification cannot proceed normally, regardless of whether the preview is valid or invalid. In contrast, when spaces between words remain intact, pre- processing of the word in the parafovea can occur. However, effective visual and orthographic processing can be carried out successfully for identity previews, but not for invalid previews (i.e., strings of random letters), and therefore under spaced conditions, initiation of identification of the correct orthographic word form might start earlier for identity than invalid parafoveal previews.

Both our EM and FRP results confirmed the prediction that the preview effect is larger for the spaced compared to the unspaced conditions. In addition, our FRP data support findings by Sheridan et al. [29] showing that the onset of preview effects is temporally delayed in unspaced compared to spaced text conditions. In their study, participants were asked to silently read sentences with inter-word spaces replaced by numbers, and parafoveal previews that could be identical to the target word or a pronounceable non-word. Sheridan and colleagues found that preview effects appeared about 20–40 ms earlier for spaced than unspaced conditions (155 ms vs. 133 ms in the survival analyses, 187 ms vs. 144 ms in simulation 3 of the study). In the present study, preview effects in the spaced condition were observed in the time-window between 70–120 ms, starting at about 95 ms, whereas preview effects in the unspaced condition started in the following time window, at about 120 ms after fixation onset. Furthermore, the authors found that the empirical data were better explained in simulations where interactions between the spacing and parafoveal preview manipulations were due to both less efficient parafoveal processing and (probably consequently) slower lexical processing. The interactions reported in the present study offer support for this hypothesis. For the unspaced conditions the preview effect was observed only between 120–300 ms, while for the spaced conditions the preview effect was found between 70–120 ms, 120–300 ms, 300–500 ms. We assumed that the time window between 70–120 ms reflected early processing of word N, which can be initiated earlier if a successful pre-processing of word N could occur in the parafovea. We also considered the time window between 300–500 ms to reflect lexical processing. Thus, the fact that we observed differences in these two time windows for the spaced, but not for the unspaced conditions might indeed indicate that parafoveal pre-processing was more efficient when spaces were kept intact, and in turn, lexical process could proceed faster in this condition relative to the unspaced condition. The lack of differences in these two time windows for the unspaced conditions might indicate that visual processing may have started at the same time and/or with the same intensity in both the identity and string unspaced conditions. A lack of word boundaries in the parafovea might have prevented effective pre-processing of low-level visual characteristics of upcoming words, as for example word length information, for both valid and invalid previews. Furthermore, this finding suggests that regardless of a valid or invalid preview, removing the spaces between words will likely produce disruption that is so great that in both circumstances word recognition might be similarly slowed down.

We note however, that for the main effect of inter-word spacing we also, unexpectedly, did not observe any significant difference between 300–500 ms. If replacing spaces with random letters produces disruption at both a parafoveal and lexical processing level, and if the time window between 300–500 ms reflects lexical processing, then we should have obtained such differences in this time window. We tentatively explain the lack of significant differences for the main effect of inter-word spacing as produced by the very comparable time course and magnitude of the neural activity in three out of four of our experimental conditions (i.e., string and identity unspaced, and string spaced conditions; see Fig 4), which might have cancelled out differences associated with processing of inter-word spacing in the parafovea.

## 5. Conclusions

In the present paper we report a co-registration experiment in which participants’ eye movements and EEG signal were recorded simultaneously. The aim of the study was to investigate the neural correlates of foveal and parafoveal processing during natural reading of sentences. In particular, we explored for the first time the neural correlates of inter-word spacing, and we examined the neural correlates of parafoveal preview during natural reading, when all the words in the sentence are manipulated.

Our eye movement results replicated the well-established findings of inter-word spacing and parafoveal preview (see [21,22] for reviews). Reading was disrupted (with longer reading times) both when spaces between words were filled by random letters, and when an invalid preview was displayed in parafovea. In addition, the FRP data showed that replacing inter-word spaces produced disruption very early after fixation onset, earlier than the effect of parafoveal previews. Taken together, the eye movement and FRP results indicate that readers made use of both low-level visual and orthographic information associated with a word extracted in the parafovea, but that spaces might be processed and influence earlier time periods within a fixation. Thus, these findings suggest that spatial selection is necessary for lexical processing to proceed normally.

Within our time windows of analyses, the FRP data examined for parafoveal preview showed both the early [1, 39–42] and, to some extent, late [40–42] *preview positivity* effects. First, we suggest that the early *preview positivity* effect might reflect not only activation of orthographic representation of a word, but disrupted or less efficient activation of the orthographic word form. Second, these findings appear to resemble more the results obtained during saccadic word-list reading experiments [40, 41], than the only other experiment conducted with reading of sentences and examining parafoveal preview type [1]. We suggest that future research might try to resolve this conundrum, that is whether the late *preview positivity* effect is observed depending on the naturalness (and possibly number of words manipulated) of the task, or whether it is a result of differences in the choice of the baseline.

Our results also provide evidence for a modulation of the preview effect based on the inter-word spacing information. When spaces are kept intact, the preview effect is larger for both eye movement and FRP measures. However, when spaces are altered and replaced by random letters, reading cannot proceed normally, and having a valid or invalid preview in parafovea is equally difficult. Under these circumstances, the only facilitation that is observed seems to be associated with the activation of the orthographic representation of the word. These results appear to provide some evidence for previous suggestions that interactive effects between inter-word spacing and parafoveal preview might be due to both efficiency of parafoveal processing and speed of lexical processing [29].
